# Using the new INTRABEAM mobile intraoperative radiotherapy system during surgery for pancreatic cancer: a case report

**DOI:** 10.1186/s13256-018-1906-6

**Published:** 2019-01-26

**Authors:** Xiaodong Song, Zili Shao, Huihong Liang

**Affiliations:** Department of Hepatobiliary Surgery, The Second Affiliated Hospital of Guangzhou Medical University, No. 250, Changgang Road, Guangzhou, 510260 People’s Republic of China

**Keywords:** Pancreatic cancer, Intraoperative radiotherapy, INTRABEAM intraoperative radiotherapy system

## Abstract

**Background:**

Pancreatic cancer is one of the most common fatal malignancies and has a poor prognosis. Surgical treatment is the most important treatment method, but there is a low rate of radical excision; moreover, the postoperative recurrence rate is high, with a local recurrence rate greater than 50%. The usefulness of intraoperative radiotherapy for pancreatic cancer has previously been examined. However, prior research was based on the traditional high-energy electron beam, which causes serious radiation toxicity. Therefore, the tumor radiation dose was limited, subsequently limiting the effect. In contrast, there is also a low-energy X-ray radiation system called INTRABEAM®. Use of INTRABEAM has been applied clinically, but the treatment effect of INTRABEAM in pancreatic cancer has not been reported.

**Case presentation:**

We present a case of a 56-year-old Chinese man with local advanced pancreatic cancer with invasion of the coeliac trunk artery and origin of the portal vein. He underwent distal pancreatectomy and splenectomy along with intraoperative radiotherapy using a portable INTRABEAM radiation system. The radiotherapy dose was 10 Gy and irradiation time was 27.4 minutes. There were no obvious postoperative complications. His abdominal pain was alleviated after surgery, and no obvious tumor recurrence has been observed in short-term follow-up.

**Conclusions:**

We believe that it is safe to apply intraoperative radiotherapy using the INTRABEAM radiation system in pancreatic cancer. This approach appears promising for further future development.

## Introduction

Pancreatic cancer is the fourth leading cause of cancer-related death [1]. Surgical resection remains the mainstay therapy; however, less than 25% of patients have cancer that is resectable at the time of diagnosis [2]. Furthermore, approximately 50% of patients experience tumor recurrence after radical operation. It is believed that this high recurrence rate is associated with minimal residual disease along the border of the intact tissues that were not excised [3, 4]; accordingly, intraoperative radiation therapy (IORT) has been used for patients with localized pancreatic cancer, including resectable or borderline resectable cases [5, 6]. IORT has been used for decades in patients with pancreatic cancer, with the goal of pain reduction and control of locoregional tumor progression [7, 8]. Formerly, IORT was performed with an electron energy source emitting a 6–20 MeV high-powered electron beam [9]: this produced promising results, but was prohibited by radiation-related complications [10]. Here, we describe a case in which we performed IORT with a low kV X-ray system called INTRABEAM® in a patient who underwent distal pancreatectomy to reduce the radiation-related complications and decrease the possibility of residual malignancy during the surgical resection. The low energy IORT system INTRABEAM provides low penetration and rapid attenuation of the radiation dose. We believed that it could eliminate the possible residual malignancies effectively and more safely than the traditional IORT.

## Case presentation

A 56-year-old Chinese man was hospitalized 2 months after the discovery of a pancreatic mass and a 1-month history of abdominal pain. He had taken no medication before hospitalization.

A physical examination after the hospitalization did not reveal any obvious abnormalities. He had a body temperature of 36.6 °C, heart rate of 99 beats per minute, blood pressure of 141/83 mmHg, respiratory rate of 20 breaths per minute, and oxygen saturation of 100%. His neurological status was normal. His family history was noncontributory. He smoked cigarettes for 20 years, but it is unknown how many cigarettes he smoked per day. He never consumed alcohol. Occupationally, he worked as an office manager.

**Fig. 1:**
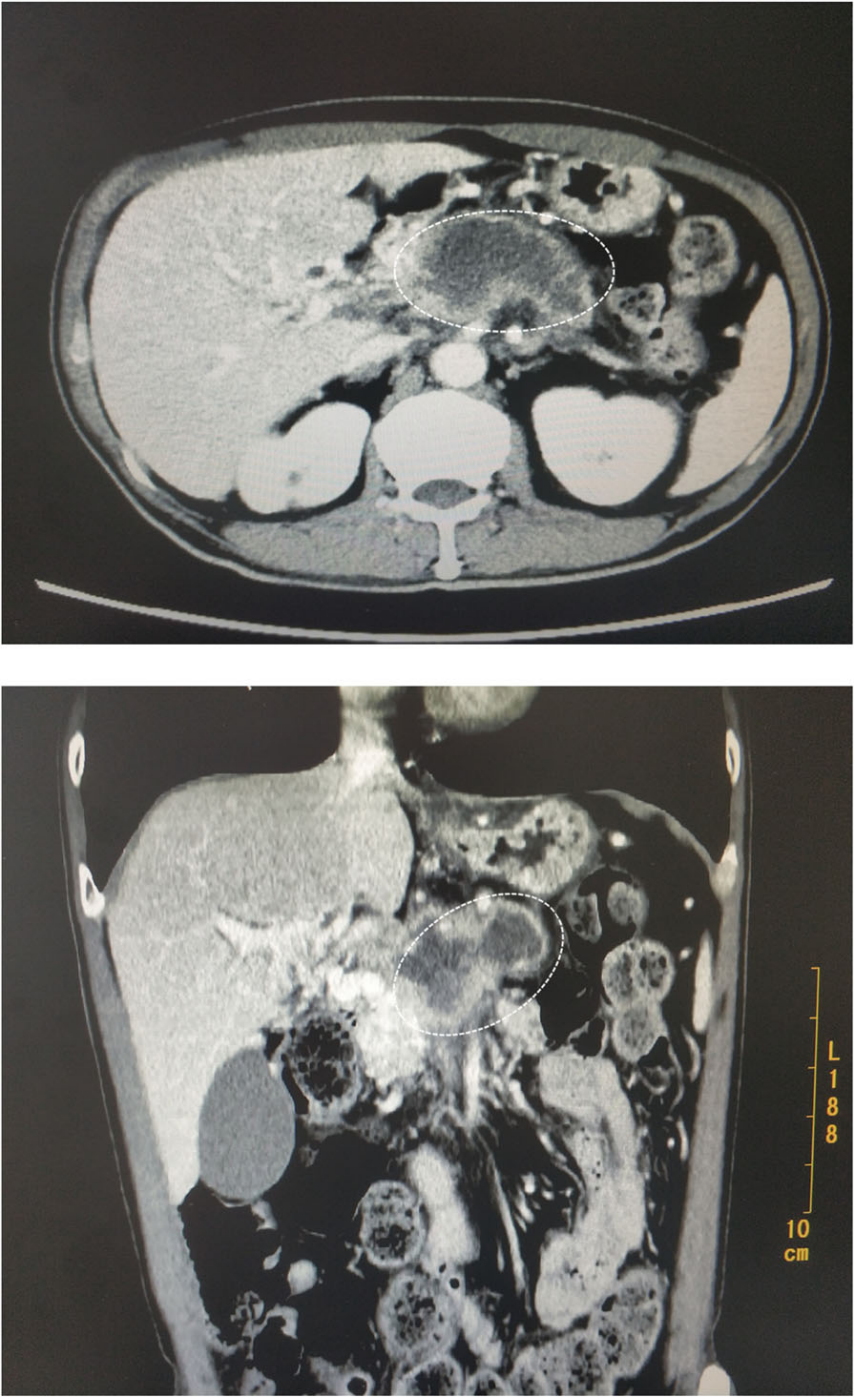
Representative computed tomography scan images. The white circle represents the location of the tumor

Laboratory test results are shown in Table 1. Blood tests revealed a high level of the CA19-9 tumor marker (1525.84 U/mL). An abdominal computed tomography scan with enhancement and vascular reconstruction revealed a space-occupying lesion in the pancreatic head and neck; the findings suggested the presence of pancreatic cancer with invasion of the hepatic artery, splenic artery, mesenteric vein, and origin of the portal vein (Fig. 1). Pathological examination of a specimen from an endoscopic ultrasound puncture biopsy revealed abnormal cells, with a morphology consistent with that of adenocarcinoma. Positron emission tomography-computed tomography revealed abnormally high fluorodeoxyglucose metabolism that was limited to the space-occupying lesion, suggesting a malignant pancreatic lesion, with atrophy of the pancreatic tail and many small nodules in the space surrounding the pancreas. He was diagnosed as having a T4N2M0 local advanced pancreatic cancer.

**Table 1:** Laboratory data on presentation

Variable	Values
Hemoglobin (g/dl)	14.0
Hematocrit (%)	38.4
White cell count (per mm^3^)	8000
Differential count (%)	
- Neutrophils	75.4
- Lymphocytes	14.6
Platelet count (per mm^3^)	273,000
Red cell count (per mm^3^)	4,700,000
Mean corpuscular volume (fl)	86
Sodium (mmol/liter)	137.6
Potassium (mmol/liter)	3.43
Chloride (mmol/liter)	91.2
Calcium (mg/dl)	2.43
Glucose (mg/dl)	82.8
Urea nitrogen (mmol/liter)	2.54
Creatinine (umol/liter)	80
Protein total (g/dl)	8.0
Albumin	4.9
Alanine aminotransferase (U/liter)	10
Aspartate aminotransferase (U/liter)	15
Bilirubin total (umol/liter)	4.7
Lactate dehydrogenase (U/liter)	188
Creatine kinase (U/liter)	118
Urine analysis	Normal
Serology for toxoplasmosis, herpes virus, CMV, EBV, rubella	Negative
CA19-9 (U/ml)	1525.84

*CMV* cytomegalovirus, *EBV* Epstein–Barr virus

**Fig. 2:**
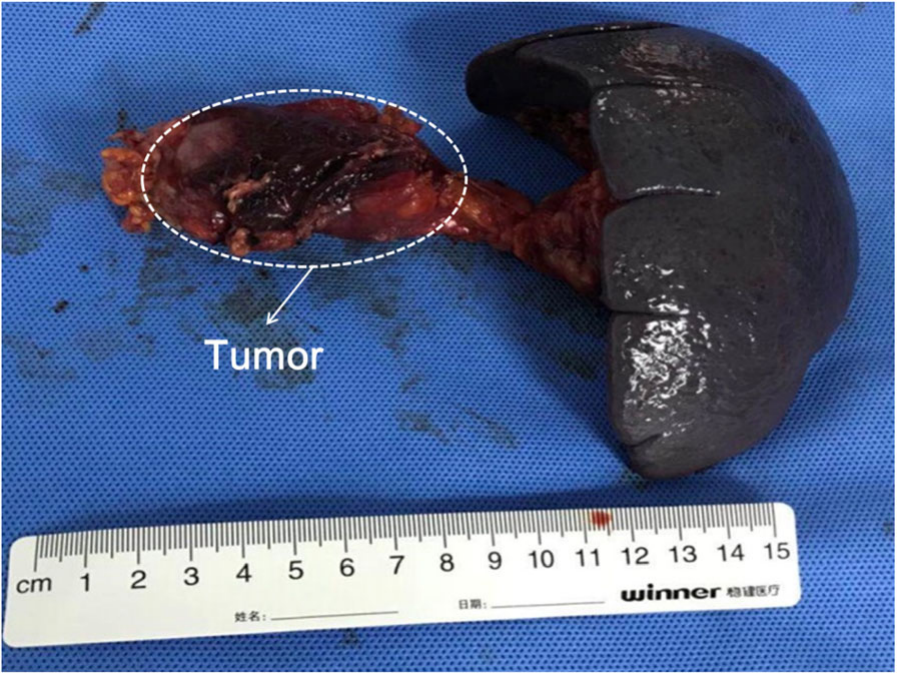
The resected gross specimen of the pancreatic cancer and spleen

Six days after admission, he underwent distal pancreatectomy and splenectomy as well as intraoperative radiotherapy (described below) under general anesthesia with tracheal intubation. A subcostal incision was made to expose his abdominal cavity, and the Kocher maneuver was subsequently performed to dissect the gastrocolic ligament and duodenal lateral peritoneum, which exposed the pancreatic tumor. The lesion was approximately 8 cm × 5 cm and had a hard texture and the tumor activity is poor (Fig. 2). Dissection and pancreatic isolation (starting from the tail and moving to the right) revealed that the tumor had invaded the superior mesenteric vein, middle colic artery, and celiac trunk artery. After isolating his spleen, the pancreatic retroperitoneum was opened, and his spleen and pancreatic tail were turned upward and isolated left to right from the posterior aspect to the superior mesenteric artery. A harmonic scalpel was used at this site to sever his pancreas, and the pancreatic duct was closed using a 4–0 Dexon suture. Local tumor remnants were observed in the posterior pancreas. The upper boundary of the remaining tumor bed reached the trunk of his abdominal cavity, the lower boundary reached his middle colic artery starting from the superior mesenteric artery, and the right boundary reached his superior mesenteric artery.

**Fig. 3:**
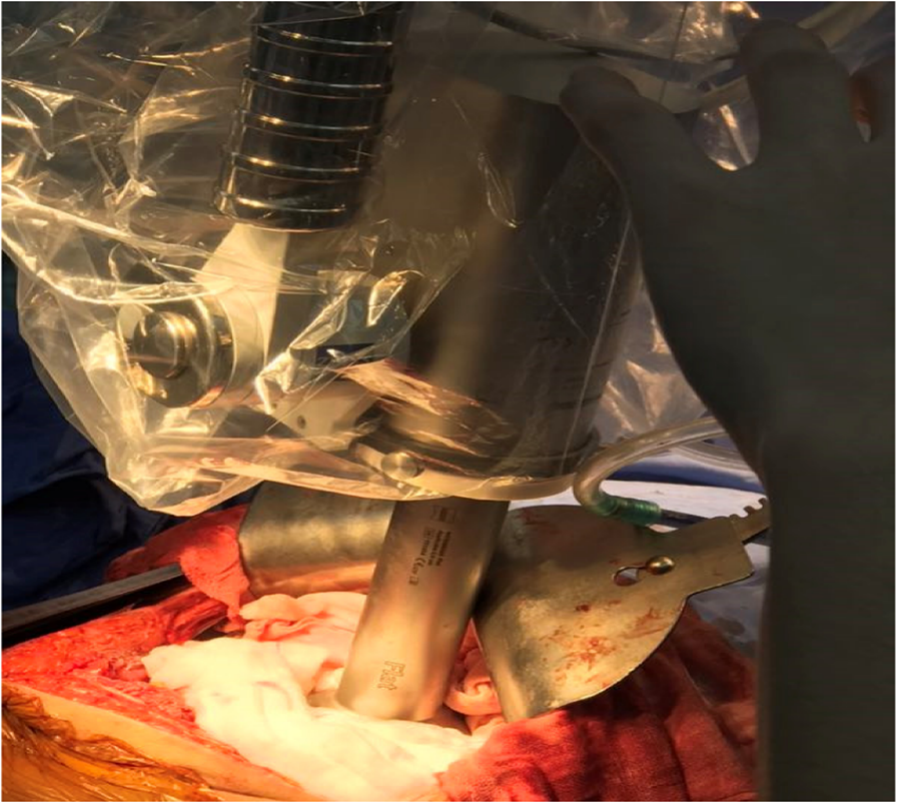
Intraoperative radiation therapy using the INTRABEAM radiation system

The INTRABEAM system was moved into the surgical field, and a 6-cm flatbed source applicator was selected based on the appearance of the tumor bed (Fig. 3). After applying an aseptic protective cover, the source applicator was placed close to the tumor bed, and our patient’s surrounding bowel and organs were protectively insulated using two layers of surgical gauze (approximate thickness 2 cm). After all personnel had left the operating room, radiotherapy was started using the following parameters: a radiotherapy dose of 10 Gy, an irradiation time of 27.4 minutes, an acceleration voltage of 50 kV, and an acceleration current of 40 μA. A drainage tube was subsequently placed at the cut edge of his pancreas, and his abdominal cavity was closed after confirming that there was no bleeding or pancreatic leakage. The total intraoperative blood loss was 200 mL.

He passed gas on postoperative day 2, and the gastric feeding tube was subsequently removed. On postoperative day 8, < 30 mL of fluid (amylase 21 U/L) had been lost via the abdominal drainage tube, which was removed before our patient was discharged on postoperative day 9. No surgery-related complications were observed, his postoperative CA19-9 level was 924.73 U/L, and his abdominal pain completely disappeared based on the numerical rating scale for cancer pain [11]. He has been followed for 6 months after surgery, and no obvious tumor recurrence has been observed.

## Discussion

Here we present a successful case of pancreatic cancer. Our patient underwent distal pancreatectomy and splenectomy along with IORT using a portable INTRABEAM radiation system. This low-energy IORT system is available to eliminate possible residual malignancies. Furthermore, it avoids the risks of severe radiotherapy-associated complications that are associated with traditional IORT.

There is a clear, increasing trend in the global incidence of pancreatic cancer, which has an extremely poor prognosis: the 5-year survival rate is < 5% [12]. Surgery is the only effective and curative treatment for pancreatic cancer, although < 25% of patients with pancreatic cancer can undergo radical surgical treatment. In addition, local recurrence develops in 50–90% of patients who undergo surgical resection, and the local recurrence rate remains high even after extensive resection. Radiotherapy is a useful palliative procedure that can provide relief from pain and other symptoms, including local pain relief in patients with unresectable tumors, which can help prolong survival and preserve quality of life. However, traditional external radiation therapy using extracorporeal irradiation can easily lead to severe complications and suboptimal overall treatment efficacy. Thus, IORT has emerged as an effective procedure for local control of unresectable tumors or tumor remnants after resection of pancreatic cancer. Unlike external radiation therapy, IORT acts directly on the treatment site, can be delivered while sparing the surrounding organs and tissues, and can deliver a large radiation dose that provides ≥ 2 times the biological effect of separate radiation doses [13], which shortens the overall treatment time.

Current domestic and international reports indicate that the Mobetron® system is the primary tool for performing IORT in cases of pancreatic cancer [14]. This system has a high electron beam energy and radiotherapeutic effects on local tumors in relatively deep locations (> 2 cm). Previous reports [15] have described its therapeutic effects on unresectable pancreatic cancers, although a relatively deep target combined with the high electron beam energy can lead to various adverse effects, such as gastrointestinal tract bleeding, bile duct fibrosis, biliary enteric anastomosis, peripheral nerve reactions, and other pancreas-associated complications. This approach can also have important effects on posterior organs and structures (for example, the vertebrae and spinal cord), which are considered major complications of IORT for pancreatic cancer. These effects include vertebral fracture, paraplegia, intraspinal hemorrhage, autonomic dysfunction, limb paralysis, and uncoordinated movement. Therefore, patients who undergo IORT for pancreatic cancer using the Mobetron system must accept a risk of developing severe radiotherapy-associated toxicities.

In the present case, we used the INTRABEAM system for IORT because it produces softer, shallower, and lower energy X-rays (30–50 kV) than the Mobetron system [16–19]. The INTRABEAM system also provides low penetration and rapid attenuation of the radiation dose [18], which are improvements that have emerged from the evolution of minimally invasive surgery. There has been rapid clinical adoption of the INTRABEAM system in recent years, with good outcomes described in studies regarding breast-conserving surgery for breast cancer [20, 21], vertebral metastasis surgery [22], and colorectal cancer surgery [23, 24]; these good outcomes are attributed to the system’s accuracy, minimally invasive nature, and low penetration depth. These characteristics allow the INTRABEAM system to protect important organs and tissues behind the pancreas and reduce the incidence of adverse effects that were associated with previous IORT systems. Moreover, given the low penetration depth, a relatively high radiation dose can be used at the clinical surface. For example, we used a radiation dose of approximately 10 Gy at the clinical surface; an effective irradiation intensity is achieved when the source applicator is < 5 mm from the target tissue. Thus, the INTRABEAM system is extremely practical for targeting residual tumor tissue after resection of pancreatic tumors. Previous studies have described doses of 10–30 Gy when the Mobetron system is used during IORT for pancreatic cancer [25, 26], although there is no established dose when using the INTRABEAM system. In the present case, we selected a relatively conservative dose of 10 Gy, and our patient did not develop any radiotherapy-related side effects, although further studies are needed to optimize the radiotherapy dose in similar cases.

The INTRABEAM system has a source applicator that comes in various shapes; this can facilitate personalized treatment based on the patient’s tumor and target sites. In the present case, our surgical exploration revealed residual tumor tissue posterior to the pancreas; this residual tumor tissue invaded the retroperitoneal blood vessels, lymph nodes, and nerve plexuses. The upper boundary reached the trunk of the abdominal cavity, the lower boundary reached the middle colic artery starting from the superior mesenteric artery, and the right boundary reached the superior mesenteric artery. Because the tumor bed was relatively uniform, with no obvious compartmentalization, and had a rough surface, we selected a flatbed source applicator that was placed close to the tumor bed. The radiotherapy was performed after protecting the surrounding bowel, and our patient experienced a good postoperative course, with recovery of gastrointestinal function on postoperative day 2. Moreover, he was able to resume eating and was free from postoperative bleeding, infection, pancreatic leakage, and other complications; in addition, he had markedly relieved pancreatic cancer-related abdominal pain. Thus, he was discharged in good general condition on postoperative day 9.

## Conclusion

In summary, based on the case described here, we conclude that using the low-energy INTRABEAM system for pancreatic cancer IORT is safe and can be applied to cases of radical resection or suspicious tumor remnants. Moreover, we conclude that the flatbed source applicator was the best fit for the tumor bed of pancreatic cancer. Although our patient experienced significant pain relief, the long-term efficacy of this approach remains unclear, and large controlled studies are needed to validate our findings. There is a need for preliminary studies for using the INTRABEAM system in pancreatic cancer, including optimal radiation dose, radiation time, and source applicator choice.

## Abbreviation

IORT: Intraoperative radiation therapy
